# Supplementary material for: Understanding how digital mental health interventions can be optimised to improve longer term sustainability: Findings from a causal mediation analysis of the CONEMO trials
Source: PLOS Glob Public Health. 2025 Jul 1;5(7):e0004537. doi: 10.1371/journal.pgph.0004537 (PMC12212485; doi:10.1371/journal.pgph.0004537)
Supplement: S1 File — (DOCX) [file pgph.0004537.s001.docx]

**S1 File: Details of mediation analyses**

**Selection of mediators**

Mediators measured only in the experimental arm are variables that are a direct consequence of receiving the DMHI and therefore not measured for participants in the control arm. Mediators were included in the final model if they were associated with another mediator, or the outcome whilst adjusting for mediator-outcome confounders (*p*<0.10).

Mediators measured in both arms of the trial were only included in the final model if they were associated with the CONEMO intervention or the outcome (*p*<0.10) whist adjusting for mediator-outcome confounders. Using the above criteria for our two types of mediators, we can capture any potential mediator that is influenced by the intervention and influences either another mediator, or the improvement in depressive symptoms.

**Mediator-outcome confounders**

Due to the randomised nature of the exposure, it is not necessary to account for confounders of the association between the exposure and the outcome. However, it is necessary to account for baseline confounders (unaffected by the intervention) that were associated with the mediator and the outcome. These mediator-outcome confounders can generate spurious correlations between the mediator and outcome when unadjusted for, potentially distorting these associations. We considered more than thirty potential baseline characteristics that are not influenced by the intervention, as potential confounders. The selection process for these confounders is described in the section on *estimation methods* below.

**Decomposition of total effect of the CONEMO intervention into direct and indirect effects**

We decomposed the total effect of the CONEMO intervention into interventional indirect effects via each of the three posited mediators and the direct effect via none of the mediators. The interventional indirect effect via a particular mediator (e.g., understanding content of the sessions without difficulty) can be interpreted as the average change in the potential outcome (improved symptoms of depression at six months) resulting from shifting the counterfactual distribution of that mediator from the exposed status (e.g., understand content of the sessions without difficulty) to the unexposed status (found content of sessions difficult to understanding), while setting each of the remaining mediators to random draws from either the exposed or unexposed group, depending on the specific decomposition. In doing so, valid inferences are not contingent on strict assumptions, such as correctly specifying the causal ordering among the mediators which are unlikely to be feasible in our current setting and can lead to incorrect inferences when violated. (1)

**Steps to estimation**

Estimation for the interventional indirect effects was based on Monte Carlo integration using a 1,000-fold expanded dataset. (15) The expanded dataset was created in four steps separately for each of the different sites.

In the first step, we fitted a model for each mediator given exposure and other predictors. Specifically, we fitted logistic (M1), ordinal (M2), and linear (M3) regression models to the observed data. Each model included a combination of predictors that were shown to be associated with the mediator of interest at the 10% level including: age, education, baseline PHQ-9 scores, baseline medication to treat a psychological problem, support from work colleagues, and use of a mobile phone. Interactions and non-linearities were explored and included if determined to be significant at the 10% level, using Stata’s post estimation command, *testparm*. The models for the pooled analyses also included a dummy variable representing the study site where interactions between study sites, and the different predictors were explored using similar criterion described above.

In the second step, the fitted mediator models were used to generate random, subject-specific Monte Carlo draws of each mediator for both the exposed and unexposed condition (i.e. counterfactual), given their observed covariate values.

In the third step, we fitted a model for the outcome, using a logistic regression model for recovery from depression, separately in the exposed and unexposed, given the mediators and mediator-outcome confounders. Any potential mediator-outcome confounder was included if it was associated with the mediator or the outcome (*p*<0.10). Models for the pooled dataset included and mediator-outcome confounder included in either of the individual sites as well as a variable to represent study site. We used a model selection criterion similar to that of the mediator models; i.e., Any relevant non-linearities and interactions were included in the outcome model if determined to be significant at the ten percent level, using the post-estimation *testparm* command in Stata.

Mediator-outcome confounders included in the São Paulo analyses include baseline medication to treat a psychological problem (yes/no), age (continuous), baseline PHQ-9 scores, number of years in education, extent to which a mobile phone is used in everyday activities (i.e. sending receiving messages, video calls, booking appointments, taking pictures, playing games, etc). Confounders in the Lima analyses were similar except for the addition of having social support from a friend or colleague (this was represented by number of times they would talk to a friend or colleague in a month).

Using the São Paulo dataset medication use for an emotional issue captured at baseline moderated BAD-SF scores measured at three-months (M3). The Lima dataset had an interaction between assigned activities completed (M2) and baseline BAD-SF scores. Continuous variables (BAD-SF at baseline and three-months (M3) and PHQ-9 at baseline) that were included in an interaction term were mean centred to reduce multicollinearity.

The pooled dataset included any mediator-outcome confounder used for the separate study sites, as well as a dummy variable representing study sites. Interactions between mediators and mediator outcome confounders were also examined for and included if the above-described criterion were fulfilled. Significant interactions were found between the following: study site and baseline medication; study site and baseline PHQ-9 scores, BAD-SF at three-months (M3) and study site; BAD-SF at three-months and medication at baseline.

In the fourth step, we used the fitted outcome model to predict the potential outcomes in the expanded dataset given the random, subject-specific draws of the mediator counterfactuals from the second step. The interventional indirect effects were then calculated as the average differences between potential outcomes under different hypothetical exposure levels.

**References**

1. Loh WW, Moerkerke B, Loeys T, et al. Heterogeneous indirect effects for multiple mediators using interventional effect models. Epidemiol Methods. 2020;9(1).
