# Supplementary material for: Understanding how digital mental health interventions can be optimised to improve longer term sustainability: Findings from a causal mediation analysis of the CONEMO trials
Source: PLOS Glob Public Health. 2025 Jul 1;5(7):e0004537. doi: 10.1371/journal.pgph.0004537 (PMC12212485; doi:10.1371/journal.pgph.0004537)
Supplement: Attachment — Submitted filename: renamed_856bd.docx [file pgph.0004537.s005.docx]

# **COMMENTS – Manuscript PGPH-D-25-00349**

Title: 'Understanding how digital mental health interventions can be optimised to improve longer term sustainability: findings from a causal mediation analysis of the CONEMO trials"

**Important note: This review pertains only to ‘statistical aspects’ of the study and so ‘clinical aspects’ [like medical importance, relevance of the study, ‘clinical significance and** **implication(s)’ of the whole study, etc.] are to be evaluated [should be assessed] separately/independently. Further please note that any ‘statistical review’ is generally done under the assumption that study specific methodological [as well as execution] issues are perfectly taken care of by the investigator(s). This review is not an exception to that and so does not cover clinical aspects {however, seldom comments are made only if those issues are intimately / scientifically related & intermingle with ‘statistical aspects’ of the study}. Agreed that** **‘statistical methods’ are used as just tools here, however, they are vital part of methodology [and so should be given due importance]. I look at the manuscript in/with statistical view point, other reviewer(s) look(s) at it with different angle so that in totality the review is very comprehensive. However, there should be efforts from authors side to improve (may be by taking clues from reviewer’s comments). Therefore, please do not limit the revision only (with respect) to comments made here.**

**COMMENTS:** Although the manuscript is flawless (rather very good / excellent), I have a little different opinion/views/observations/concerns or rather questions & suggestions regarding very few issues which are given below:

Firstly, I noted that your ABSTRACT is well drafted (in my opinion), but is ‘assay type’. It is preferable [refer to item 1b of CONSORT checklist 2010: Structured summary of trial/study design, methods, results, and conclusions] to divide the ABSTRACT with small sections like ‘Objective(s)’, ‘Methods’, ‘Results’, ‘Conclusions’, etc. which is an accepted practice of most of the good/standard journals [including this one, though the ‘Guidelines to Authors’ may/did not specify an Abstract format, it is desirable]. It will definitely be more informative then, I guess, whatever the article type may be. Since separate paragraphs (presently) summarize the manuscript, you just have to give different headings to paragraphs in abstract [very easy indeed].

Secondly, please confirm the level of measurement of all the variables. Though few variables are continuous in appearance they are likely to yield data that are in [at the most] ‘ordinal’ level of measurement and not in ratio level of measurement for sure [for example: PHQ-9 or BAD-SF]. Then application of suitable non-parametric (or distribution free) test(s) is/are indicated/advisable [even if distribution may be ‘Gaussian’ (also called ‘normal’)]. Agreed that there is/are no non-parametric test(s)/technique(s) available to be used as alternative in all situation(s), but should be used whenever/wherever they are available. Therefore, in short use suitable non-parametric test(s)/technique(s) while dealing with data that are in ‘ordinal’ level of measurement even if [despite that] the distribution may be ‘Gaussian’.

For ordinal variables descriptive summary statistic such as (Mean, SD) may not be a correct representation. In case of few/At some-times ‘pooling’ of different samples may be questionable [for example: ‘number of assigned activities completed’ or ‘Years of education’ or ‘Medication to treat emotional problem’ or ‘Have friends to contact if needed’ as given in table-1].

As pointed out in ‘important note’ above “This review pertains only to ‘statistical aspects’ of the study and so ‘clinical aspects’ should be assessed separately/independently [one should carefully consider/look at the clinical implications of the study]. In my opinion, to make this article [more] acceptable (which is quite possible and easy), a small amount of re-vision (re-drafting) may be needed. Therefore, ‘Minor revision’ is recommended.
