# Supplementary material for: Understanding how digital mental health interventions can be optimised to improve longer term sustainability: Findings from a causal mediation analysis of the CONEMO trials
Source: PLOS Glob Public Health. 2025 Jul 1;5(7):e0004537. doi: 10.1371/journal.pgph.0004537 (PMC12212485; doi:10.1371/journal.pgph.0004537)
Supplement: Attachment — Submitted filename: Rebuttal letter final.docx [file pgph.0004537.s007.docx]

**Rebuttal letter**

**Reviewer #1: Important note: This review pertains only to ‘statistical aspects’ of the study and so ‘clinical aspects’ [like medical importance, relevance of the study, ‘clinical significance and implication(s)’ of the whole study, etc.] are to be evaluated [should be assessed] separately/independently. Further please note that any ‘statistical review’ is generally done under the assumption that study specific methodological [as well as execution] issues are perfectly taken care of by the investigator(s). This review is not an exception to that and so does not cover clinical aspects {however, seldom comments are made only if those issues are intimately / scientifically related & intermingle with ‘statistical aspects’ of the study}. Agreed that ‘statistical methods’ are used as just tools here, however, they are vital part of methodology [and so should be given due importance]. I look at the manuscript in/with statistical viewpoint, other reviewer(s) look(s) at it with different angle so that in totality the review is very comprehensive. However, there should be efforts from authors side to improve (may be by taking clues from reviewer’s comments). Therefore, please do not limit the revision only (with respect) to comments made here.**

Thank you for this clarification.

**COMMENTS: Although the manuscript is flawless (rather very good / excellent), I have a little different opinion/views/observations/concerns or rather questions & suggestions regarding very few issues which are given below:**

Thank you for your positive and encouraging assessment! Below, we have endeavoured to carefully address each important comment you’ve raised in turn.

**Firstly, I noted that your ABSTRACT is well drafted (in my opinion), but is ‘assay type’. It is preferable [refer to item 1b of CONSORT checklist 2010: Structured summary of trial/study design, methods, results, and conclusions] to divide the ABSTRACT with small sections like ‘Objective(s)’, ‘Methods’, ‘Results’, ‘Conclusions’, etc. which is an accepted practice of most of the good/standard journals [including this one, though the ‘Guidelines to Authors’ may/did not specify an Abstract format, it is desirable]. It will definitely be more informative then, I guess, whatever the article type may be. Since separate paragraphs (presently) summarize the manuscript, you just have to give different headings to paragraphs in abstract [very easy indeed].**

We did not include a structured abstract with separate headings as per the journal guidance that can be found here: <https://journals.plos.org/globalpublichealth/s/submission-guidelines#loc-parts-of-a-submission>

**Secondly, please confirm the level of measurement of all the variables. Though few variables are continuous in appearance they are likely to yield data that are in [at the most] ‘ordinal’ level of measurement and not in ratio level of measurement for sure [for example: PHQ-9 or BAD-SF]. Then application of suitable non-parametric (or distribution free) test(s) is/are indicated/advisable [even if distribution may be ‘Gaussian’ (also called ‘normal’)]. Agreed that there is/are no non-parametric test(s)/technique(s) available to be used as alternative in all situation(s), but should be used whenever/wherever they are available. Therefore, in short use suitable non-parametric test(s)/technique(s) while dealing with data that are in ‘ordinal’ level of measurement even if [despite that] the distribution may be ‘Gaussian’.**

Thank you for highlighting this issue. We apologize for a small error in the labelling of the different variables in Tables 1 and 2 that may have contributed to your confusion. Per your comment, we have now corrected this error. For M1 we have clarified that this mediator is the ‘Number (proportion) of participants who understand content of sessions. For M2 we have clarified that it measures the number (proportion) of assigned activities completed: no activities; 1-10 activities; 11-27 activities. For M3 we have clarified that we are referring to the mean/SD for levels of activation/avoidance. We have also clarified some of the labels for our mediator-outcome variables.

We agree that applying appropriate non-parametric tests might be useful. However, we have opted not to perform any statistical tests in Table 1 and Table 2. This aligns with current guidance to avoid categorical statements of statistical significance (e.g., "statistically significant" vs. "not significant"), which can lead to binary, oversimplified thinking or distort scientific interpretation by focusing solely on variables that meet arbitrary thresholds. Instead, we are simply presenting descriptive proportions for categorical variables across different studies so readers can visually assess any differences that they may deem clinically meaningful. We are also presenting means (and SDs) of continuous variables across different studies for readers . Furthermore, when estimating the total causal effect and indirect effects for the different mediators we are using the non-parametric bootstrap to construct confidence intervals that capture important information about effect magnitude and sampling variability, crucial for better informing scientific conclusions (please see Table 3).

**For ordinal variables descriptive summary statistic such as (Mean, SD) may not be a correct representation. In case of few/At some-times ‘pooling’ of different samples may be questionable [for example: ‘number of assigned activities completed’ or ‘Years of education’ or ‘Medication to treat emotional problem’ or ‘Have friends to contact if needed’ as given in table-1].**

As we describe above, we feel that an error with the description of some variables may have caused some confusion. We have now corrected the labelling in both Table 1 and Table 2.

With regards to the pooling different samples, we are presenting proportions for categorical variables and means (SD) for continuous variables using data from both Lima and Sao Paulo. In doing so we hope readers are able to compare how these estimates differ to the estimates from the data for the individual trials. This is important as our mediation analysis uses this data to calculate indirect effects for the separate trials, as well as using the pooled dataset.

**As pointed out in ‘important note’ above “This review pertains only to ‘statistical aspects’ of the study and so ‘clinical aspects’ should be assessed separately/independently [one should carefully consider/look at the clinical implications of the study]. In my opinion, to make this article [more] acceptable (which is quite possible and easy), a small amount of re-vision (re-drafting) may be needed. Therefore, ‘Minor revision’ is recommended.**

Many thanks for your positive review. We have re-read the draft of the manuscript and made edits you recommended as well as completing some copy editing.

**Reviewer #2: The manuscript is a thorough investigation on how digital mental health interventions (DMHIs) can potentially be optimized for long-term impact. It provides valuable insights into understanding the role of different mediators such as ease of understanding session content, activity completion, and behavioural activation in improving depression outcomes.
The paper explains the structure of the CONEMO intervention well, describing how the app sessions are delivered over six weeks and how mediators like the number of activities completed (M2) and behavioural activation (M3) are measured through established instruments like the BADS-SF. Additionally, the inclusion of both individual trial results and pooled analysis emphasizes the robustness of the findings across different settings (São Paulo and Lima) which is good for the generalizability of your conclusions.**

Many thanks for your positive review of our mediation analysis including the pooling of data which we agree improves the robustness of our findings.

**A few areas could benefit from additional clarification or expansion:
Generally, check the citation style of the journal, it is ‘[ ]’ instead of ‘( )’ brackets.**
We have now reviewed the manuscript and corrected all references replace ‘()’ with []’

**Methods**

**Line 208 to 212: Consider providing justification for the categorisation of the mediators.**

We originally described this in the supplementary material but have now moved this to the main paper on page 8 of the revised manuscript, where we write:

“*Causal mediation analysis using interventional (in)direct effects is grounded in a potential outcomes framework. This approach requires probabilities of the mediators to be simulated in both the exposed and unexposed (counterfactual) population using predictive models. For mediators measured in both arms of the trials, observed values in the experimental arm were used to simulate probabilities for the exposed population, and the observed values in the control arm were used to simulate probabilities for the unexposed population. For mediators measured in the experimental arm only, the values were categorised so that an “unexposed” value of zero was defined for those who were assigned to the experimental arm but were not exposed to the mediator of interest (i.e., did not complete any of the assigned activities; similar to those in the control arm who were unexposed).* A full description of the potential mediators and selection criterion can be found in Supplement 1.”

**Line 212 to 257: Consider clarifying the theoretical reasoning behind choosing these mediators and how they are interrelated.**
*For M1 we now write on pages 8/9:* *We theorised that participants who found the DMHI difficult to use, would also have difficulty completing assigned activities, potentially not improving levels of activation and avoidance. In turn, this would influence whether participants experienced a reduction in symptoms of depression.*

For M2 we now write on page 9*: We theorised that participants who completed activities that were self-selected to improve levels of enjoyment, would help to improve levels of activation and avoidance, potentially reducing symptoms of depression.*

For M3 we write on page 10**:** *We theorised that participants who completed a higher proportion of activities they found enjoyable and meaningful (M2) would have higher levels of activation and avoidance captures with the BAD-SF instrument (M3).*

**Discussion
You have appropriately acknowledged potential issues such as recall bias for the first mediator and the limitations in categorizing the activity completion variable. Consider expanding the discussion on how these limitations may affect the interpretation of the results and possible strategies to mitigate these effects in future studies.**

We have now updated the section on limitations on pages 20/21 to the following**:** *Given that our first mediator was collected up to a month and a half after the intervention took place, there is a chance of recall bias. For instance, participants who had severe symptoms of depression may feel this has to do with the DMHI and report that it was difficult to use, compared to someone with less severe symptoms of depression. This bias could increase the proportion of the total effect mediated through our first mediator. Future DMHI should consider capturing this information electronically as soon as the session is complete. Lastly, our second mediator representing the number of activities completed was captured using a categorical variable (no activities, 1-10 activities, 11-27 activities). Categorising this mediator could have led to diluting the indirect effect and inflating the direct effect.[**8**] There is another issue with the second mediator, where our unexposed category had a limited number of participants, leading to wide confidence intervals. However, when we pooled the datasets, this limitation was largely overcome. Nevertheless, it is possible the indirect effect through number of activities completed may be underestimated.*

**The finding suggests that ensuring clear, simple session content can lead to higher rates of activity completion and greater levels of behavioural activation, ultimately resulting in improved mental health outcomes. More discussion on how these insights might be applied practically to enhance digital interventions at scale could make the paper even more impactful.**

We have now updated the discussion on page 21 to the following: *Future interventions should consider findings from this analysis when designing digital interventions to treat depression. In particular, careful consideration needs to be given to ensure the instructions in the DMHI are easy to understand for the most vulnerable. Co-design approaches involving people with lived experience as well as participants who are more likely find the digital app difficult to use (e.g., older participants with severe symptoms of depression) could help to ensure session content is easy to understand. Adequate human support could also be used to answer queries so as not to discourage patients from participation as well as to encourage patients to complete all activities. Importantly, although human support has been identified as an key feature for digital interventions in improving clinical effectiveness.[**28**] too much will inflate the costs of digital interventions, making them difficult to sustain in poorly resourced settings.*
